# A Hospital Social Evening

**Published:** 1923-03

**Authors:** 


### A HOSPITAL SOCIAL EVENING.

THE success which attended the last of the Social and Scientific Evenings held at the Middlesex Hospital on February 9 must have been very inspiriting to the promoters. The scheme is the outcome of what may be described as " a new movement " in connection with hospital work, a desire to secure for the London institutions the same personal relationship with their supporters as exists in many provincial towns and country places. The following have formed a group: The Hospital for Paralysis and Epilepsy, the National Hospital for Diseases of the Heart, Queen Charlotte's Lying-in Hospital, Royal National Orthopædic Hospital, Samaritan Free Hospital for Women, West End Hospital for Nervous Diseases, and the Western Ophthalmic Hospital, with the Middlesex as an organising centre. By means of these evenings the hospitals hope to get into closer touch with the employers and employés who yearly give of their substance to the support of any individual institution.

The working of the scheme is very simple. Notification of the date of an evening when the hospital will be open to the inspection of its friends is given and printed invitation tickets sent to any who make application. At the first evening 30 visitors were present; at the fifth and last of the current series 796 persons asked to be allowed to come. A large proportion of these were young men and young women—the former largely predominated—who are employed in the large stores, shops and factories in the neighbourhood. During the evening they visited the Operating Theatres and the Radiological Department, where many found the marvels of X-ray, as showing them the bones in their own hands, of special interest. The Physics Laboratory, Museum, and the Board Room with its historical exhibits were also thrown open. In each case members of the medical or surgical staff kindly gave their services to explain and answer questions. There were short lantern talks on radium, on minute marvels, on Life in the Highlands, and moving pictures were shown by the new Kine Reflex method. Organ recitals were held in the Chapel at intervals and a string quartette and a delightful vocalist gave a concert in the Students' Memorial Room, where coffee and cakes were served throughout the evening. The waitresses consisted mainly of volunteers of the clerical staff of the hospital.

The Controller, Major J. R. Ainsworth Davies, M.A., M.Sc., F.C.R., F.Z.S., and his able assistant, Mrs. Philip Smith, are to be congratulated warmly on the successful initiation of a new development in hospital life which, it is hoped, will be followed next winter by other institutions in London and elsewhere.

